# Functionally reciprocal mutations of the prolactin signalling pathway define hairy and slick cattle

**DOI:** 10.1038/ncomms6861

**Published:** 2014-12-18

**Authors:** Mathew D. Littlejohn, Kristen M. Henty, Kathryn Tiplady, Thomas Johnson, Chad Harland, Thomas Lopdell, Richard G. Sherlock, Wanbo Li, Steven D. Lukefahr, Bruce C. Shanks, Dorian J. Garrick, Russell G. Snell, Richard J. Spelman, Stephen R. Davis

**Affiliations:** 1 Livestock Improvement Corporation, Cnr Ruakura and Morrinsville Roads, Newstead, Hamilton 3240, New Zealand; 2 School of Biological Sciences, University of Auckland, 3A Symonds Street, Auckland 1010, New Zealand; 3 Unit of Animal Genomics, GIGA-R, Faculty of Veterinary Medicine, University of Liège (B34), Liège B-4000, Belgium; 4 Department of Animal, Rangeland and Wildlife Sciences, Texas A&M University-Kingsville, MSC 228, Kingsville, Texas 78363-8202, USA; 5 Department of Agriculture and Environmental Science, Lincoln University, 820 Chestnut Street, Jefferson City, Missouri 65101, USA; 6 Department of Animal Science, Iowa State University, 225 Kildee, Ames, Iowa 50011-3250, USA

## Abstract

Lactation, hair development and homeothermy are characteristic evolutionary features that define mammals from other vertebrate species. Here we describe the discovery of two autosomal dominant mutations with antagonistic, pleiotropic effects on all three of these biological processes, mediated through the prolactin signalling pathway. Most conspicuously, mutations in prolactin (*PRL)* and its receptor *(PRLR)* have an impact on thermoregulation and hair morphology phenotypes, giving prominence to this pathway outside of its classical roles in lactation.

## Abstract (web summary)

The hormone prolactin is a known modulator of mammalian lactation and hair growth. Here, the authors describe two dominant mutations in bovine prolactin and its receptor, demonstrating antagonistic effects on these traits and highlighting a role for this pathway in sweat gland function and thermoregulation.

## Graphical abstract

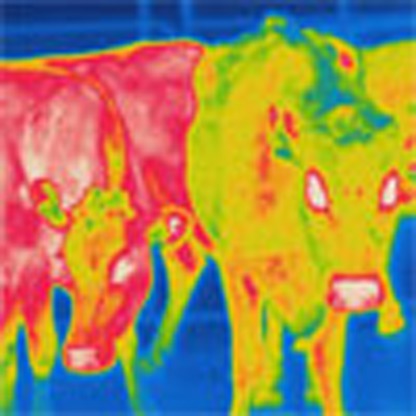

Hallmark characteristics of mammals include the secretion of milk, the development of body hair and the homeothermic regulation of body temperature. These latter two processes have clear physiological links, with one of the key functions of body hair being to insulate the endothermic animal. Lactation also shares some common biology with these processes, where similarities in the structure and function of mammary, sweat and sebaceous glands has led to the hypothesis that mammary glands evolved from a pilosebaceous apocrine structure in the skin^1^. The literature describing the cellular and molecular physiology of each of these phenomena is vast, and in the case of mammary and hair follicle biology, these processes are known to be broadly regulated by a range of hormones including oestrogen^2^,^3^,^4^, testosterone^4^,^5^,^6^, growth hormone^7^,^8^, prolactin^9^,^10^ and others^11^.

In 2011 we identified a spontaneous, dominant genetic syndrome in *Bos taurus*, presenting as a collection of unusual phenotypes including lactation failure, excessively ‘hairy’ pelage and thermoregulatory dysfunction. We report mapping of the mutation for this syndrome, and further report identification of a novel, phenotypically reciprocal mutation in the same molecular pathway, defining the slick-coated, thermotolerant characteristics of the Senepol breed of cattle.

## Results

### A novel pleiotropic syndrome in dairy cattle

**Figure 1:**
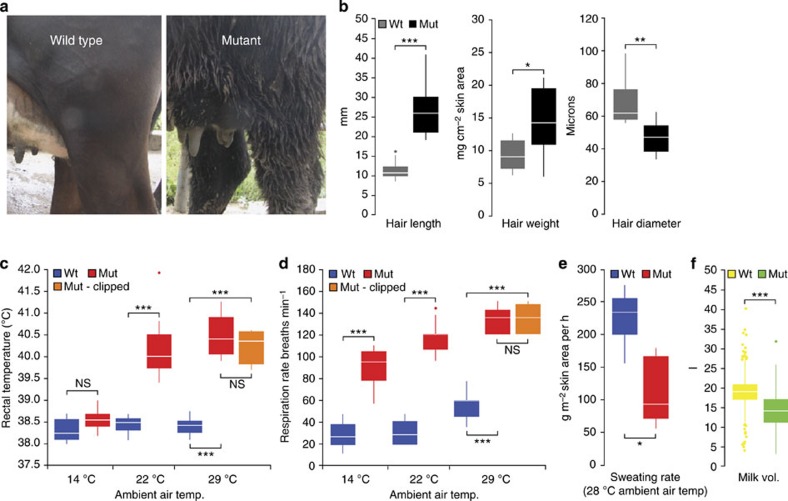
Phenotypic characteristics of hairy syndrome cattle. (**a**) Photograph showing coat differences between wild-type and mutant half-sibs, with muddy coat due to wallowing behaviour typical of affected animals. (**b**) Hair morphology differences between mutant (*N*=12) and wild-type (*N*=12) half-sibs. (**c**,**d**) Heat stress response phenotypes of mutant (*N*=12) and wild-type (*N*=12) half-sibs measured at different ambient temperatures. Responses of twelve wild-type, seven mutant and five clipped mutants also indicated. (**e**) Sweating rate contrast between mutant (*N*=6) and wild-type (*N*=6) cows. (**f**) Differences in milk volumes between wild-type (*N*=740) and mutant (*N*=77) half-sibs. These differences underestimate the extent of lactation effects since at least 25% of mutant animals failed to initiate lactation. Box plots define the median, upper and lower quartiles for the various phenotypes, with whiskers representing the furthest data points within 1.5 × of the interquartile range, and outlier samples indicated beyond this range. *P<0.05, **P<0.001, ***P<0.0001 (two-sided *t*-tests, Bonferroni-adjusted).

As part of routine animal screening in a large dairy cattle-breeding programme in New Zealand, we identified a dominant genetic syndrome that had been rapidly propagated through the population through widespread use of semen representing a bull and his son. Animals within the pedigree (*N*>6,000) segregated for abnormally long and ‘hairy’ coats, symptoms of heat stress including increased respiration rates and the tendency to wallow in mud and drinking troughs, and major defects in lactation. Detailed examination of 12 affected and 12 control females showed that hair was more slender (two-sided *t*-test, *P*=1.4 × 10^−4^) and approximately twice as long in affected animals (two-sided *t*-test, *P*=1.4 × 10^−7^; Fig. 1a,b; Supplementary Fig. 1). There was also an increase in hair mass per cm^2^ of skin area in affected animals (two-sided *t*-test, *P*=0.012; Fig. 1b), although this effect was not apparent when adjusted for hair diameter and length (two-sided *t*-test, *P*=0.226), suggesting a similar density of hair of increased fibre weight.

At an ambient temperature of 22 °C (thermoneutral for *Bos taurus*), rectal temperatures were elevated in affected animals compared with controls (two-sided *t*-test, *P*=1.3 × 10^−8^; Fig. 1c). Heart rates were not significantly different between groups (two-sided *t*-test, *P*=0.149; Supplementary Fig. 2); however, respiration rates were approximately four times greater in affected individuals (two-sided *t*-test, *P*=2.6 × 10^−14^; Fig. 1d). These effects were reproducible over multiple time points and days (Supplementary Table 1 and Supplementary Fig. 2). Since this heat stress response could have been partly attributable to increased hair length, five of twelve affected animals were clipped to approximate the coat lengths of controls (Supplementary Fig. 1). This had no effect on body temperatures or respiration rates (Fig. 1c,d). Since sweating and panting are the primary modes of active heat loss in cattle, we next assessed the sweating rates of six affected and six control animals. At an ambient indoor temperature of 28 °C, control cattle produced twice the weight of sweat compared with hairy animals (two-sided *t*-test, *P*=0.001; Fig. 1e), implicating sweat gland dysfunction as the likely source of thermoregulatory failure.

Affected females also failed to lactate or produced markedly less milk (two-sided *t*-test, *P*=3.7 × 10^−21^; *N*=817; Fig. 1f). Although heat stress may have contributed to these effects, they appeared to be a primary feature of the syndrome, since >95% of lactation records from affected animals were measured during spring at cool to moderate temperatures (September to November 2013; mean daily temperature <16 °C for all geographic regions). Further, the influence on milk yield was similar when comparing farms between the North Island and South Island (Supplementary Table 1), where the mean daily temperature from September to November was 14.1 and 11.6 °C for each island, respectively.

### Mapping the ‘hairy’ mutation

**Figure 2:**
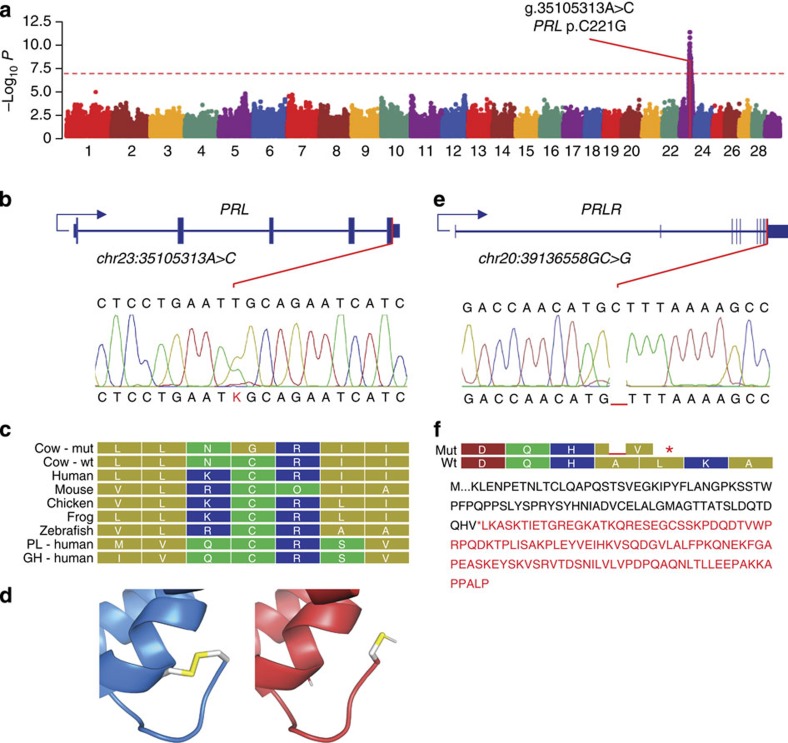
Mapping and bioinformatic characterization of *PRL* and *PRLR* mutations. (**a**) Manhattan plot showing the *hairy* locus on chromosome 23, with significance plotted on the *y* axis, and chromosome number and position indicated on the *x* axis. (**b**,**e**) Graphics depicting *PRL* and *PRLR* gene structures, showing locations of the respective p.Cys221Gly and *PRLR* p.Leu462* mutations and representative Sanger sequence traces. (**c**) ClustalW alignment showing conservation of the prolactin Cys221 residue in five vertebrates, and in human placental lactogen and growth hormone (residues coloured by polarity). (**d**) Disruption of the C-terminal disulphide bridge because of p.Cys221Gly, modelled on the 3D structure of human prolactin (1RW5.pdb). (**f**) 200 C-terminal amino acids of *PRLR*, with truncated residues because of the p.Leu462* mutation indicated in red.

To identify the ‘*hairy*’ locus and mutation, we undertook genome-wide transmission disequilibrium testing using 628,278 single-nucleotide polymorphisms (SNPs) in 22 nuclear trios and 55 half-sib offspring of the two founder sires. This analysis revealed a single significant locus on chromosome 23 (sib-transmission/disequilibrium test, *P*=1.7 × 10^−12^; Fig. 2a), with the most highly associated SNP (rs110103404) mapping within 0.5 Mbp of the *MIR2284C*, *HDGFL1* and *PRL* genes. Given the key roles of prolactin signalling in mammary differentiation^12^, and hair follicle growth and cycling^13^, we considered *PRL* as a candidate gene at the *hairy* locus. Sanger sequencing of *PRL* in both sires revealed a single candidate mutation that was not present in the National Center for Biotechnology Information (NCBI) database for short genetic variations (dbSNP), or our own whole-genome sequence database of 554 contemporary animals (ss1067289409; chr23:35105313A>C; Fig. 2b). This nonsynonymous SNP in exon 5 encodes a p.Cys221Gly substitution highly conserved across vertebrates and other structurally related hormones, disrupting one of three disulphide bonds defining the three-dimensional (3D) structure of mature prolactin hormone (Fig. 2c,d). To assess the candidacy of other mutations at this locus, we then conducted genome sequencing of the two founder sires. Filtering all previously unobserved variants assuming a dominant, heterozygous genetic model yielded only seven variants chromosome-wide, only one of which mapped to exonic sequence, being the same *PRL* mutation discovered using our candidate-led approach (Supplementary Table 2). The p.Cys221Gly variant was then genotyped in 2,205 progeny of the two sires, demonstrating complete concordance between affected (*N*=1,045) and unaffected (*N*=1,160) individuals.

### A candidate pathway for thermoregulatory mutations in other cattle

**Figure 3:**
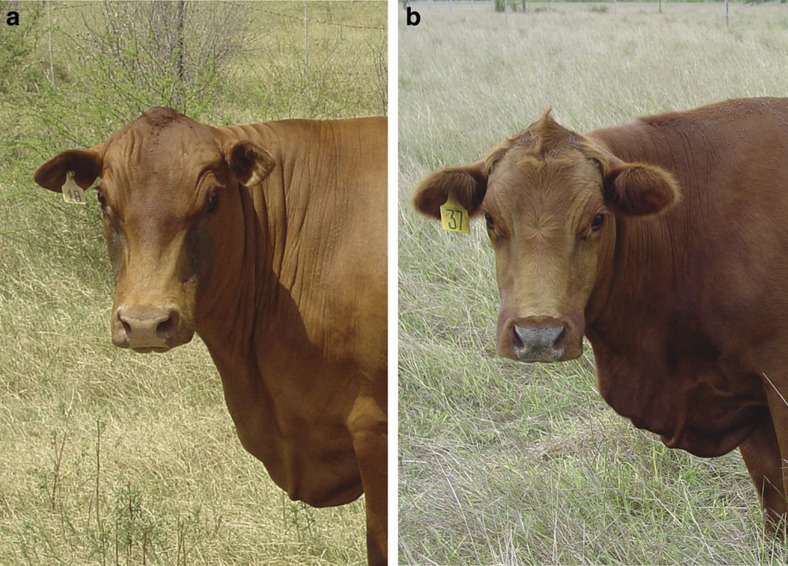
‘Slick’ coat type. Photographs contrasting slick and nonslick Senepol crossbreeds. The animal pictured on the left (**a**) carries the *PRLR* p.Leu462* mutation and is a three-way cross of Tuli (0.5), Senepol (0.25) and Red Angus (0.25); the animal on the right (**b**) is wild-type and contains Senepol (0.375), Red Angus (0.25), Beefmaster (0.1875) and Simmental (0.1875) ancestry. Pictured animals are representative of the crossbreeds used for genetic analysis of the *slick* locus, representing coat scores of 1 and 4, respectively.

With genetic data from the hairy pedigree strongly supporting the causative status of the *PRL* p.Cys221Gly variant, we next contemplated whether coat conformation and heat tolerance in other cattle might be influenced by other mutations in prolactin signalling pathways. The individual coat types of domesticated bovine breeds vary widely, with yak breeds (*Bos grunniens*) selected for hair length and cold tolerance, and short-haired cattle such as zebu (*Bos indicus*) selected for hot, tropical environments. Most *Bos taurus* breeds are temperate-adapted; however, Senepol is one of a small number of breeds that is heat-tolerant, ostensibly due to their unusually short, ‘slick’ coats (Fig. 3). This trait is thought to be determined by a single, dominant mutation^14^, with the ‘*slick*’ locus spanning a region on chromosome 20 (refs 15, 16, 17) that includes the prolactin receptor (*PRLR*). We thus considered *PRLR* as a positional candidate gene for the slick coat phenotype, and sequenced *PRLR* in a purebred Senepol sire. We identified a single homozygous frameshift mutation not present in dbSNP or our sequence database, consisting of a single base deletion in exon 10 that introduces a premature stop codon (p.Leu462*) and loss of 120 C-terminal amino acids from the long isoform of the receptor (ss1067289408; chr20:39136558GC>G; Fig. 2e,f).

### Association analysis at the *slick* locus

We next typed the *PRLR* p.Leu462* mutation in four purebred Senepol sires whose progenies were known to segregate for slick coat type, with the mutation confirmed as heterozygous in these animals. We then genotyped a collection of 82 highly crossbred cattle containing 0.5–0.0625 Senepol ancestry. Coat length was scored on a quantitative scale (where 1=slick, 4=long), since polygenic background effects in crossbreeds can result in slight increases in hair length over that seen in purebred Senepol animals^14^. The mutation was highly associated with coat length in these animals (genotypic test assuming dominance, *P*=7.3 × 10^−20^), and when considered as a binary trait comprising 42 cases and 40 controls (1=slick, >1=not slick), the mutation segregated in 79 of 82 individuals (genotypic test assuming dominance, *P*=4.7 × 10^−17^). This proportion of nonsegregating animals was similar to that reported for slick-coat phenotype transmission rates in other crossbreeds^14^, and for the two nonslick animals that carried the *PRLR* p.Leu462* mutation, both had quantitative scores of ‘2’ (Supplementary Table 3), supporting a hypothesis of phenotype ambiguity or misassignment in these animals. Haplotype-based analysis was then conducted using 25 Illumina SNP50 BeadChip SNPs in a 1-Mbp consensus *slick* interval reported in independent analyses of Senepol^16^ and Senepol crossbreeds^17^. This analysis revealed maximum significance for a 229-kb haplotype block bearing the p.Leu462* mutation (two-sided *t*-test assuming dominance, *P*=2.4 × 10^−19^), with the corresponding ancestral-allele haplotype unassociated with coat length (Supplementary Tables 4 and 5). Notably, haplotypes of the third nonsegregating animal did not share an obvious lineage with the 229-kb contiguous block found in all other slick-coded animals, making the existence of an alternative, hidden causative mutation shared by all slick-coded animals unlikely. These data suggested that the *PRLR* p.Leu462* mutation, or some other, unknown variant carried by the same haplotype was responsible for the slick-coat phenotype.

### Exome sequence analysis

To look for alternative mutations at the *slick* locus, we next obtained exome sequence data from 115 animals representing Senepol, Angus, Belgian Blue, Brahman, Charolais, Holstein Friesian, Jersey, Nelore, Simmental and Yak breeds. Restricting analysis to the 1-Mbp *slick* interval used for haplotype testing, and filtering to nonreference variants that were present in all Senepol, but absent in all other breeds yielded only the *PRLR* p.Leu462* variant. Our exome sequence panel included *Bos indicus* breeds that are also short-coated and heat tolerant (Brahman and Nelore). Although the short coat of indicus cattle is not reported as a segregating trait, it is conceivable that Senepol coat type was derived from this species, given the recent proposal that Senepol contains minor proportions of indicus ancestry^16^. As an alternative analysis, we pooled Senepol, Nelore and Brahman animals, and filtered to all nonreference variants that were shared by these breeds, but were absent in all others. This yielded no variants in the 1-Mbp interval of interest, suggesting that Senepol coat type did not arise through introgression of fixed *Bos indicus* alleles, and further supporting the *PRLR* p.Leu462* variant as the only plausible causative mutation.

### Histological and molecular characteristics of hairy and slick cattle

**Figure 4:**
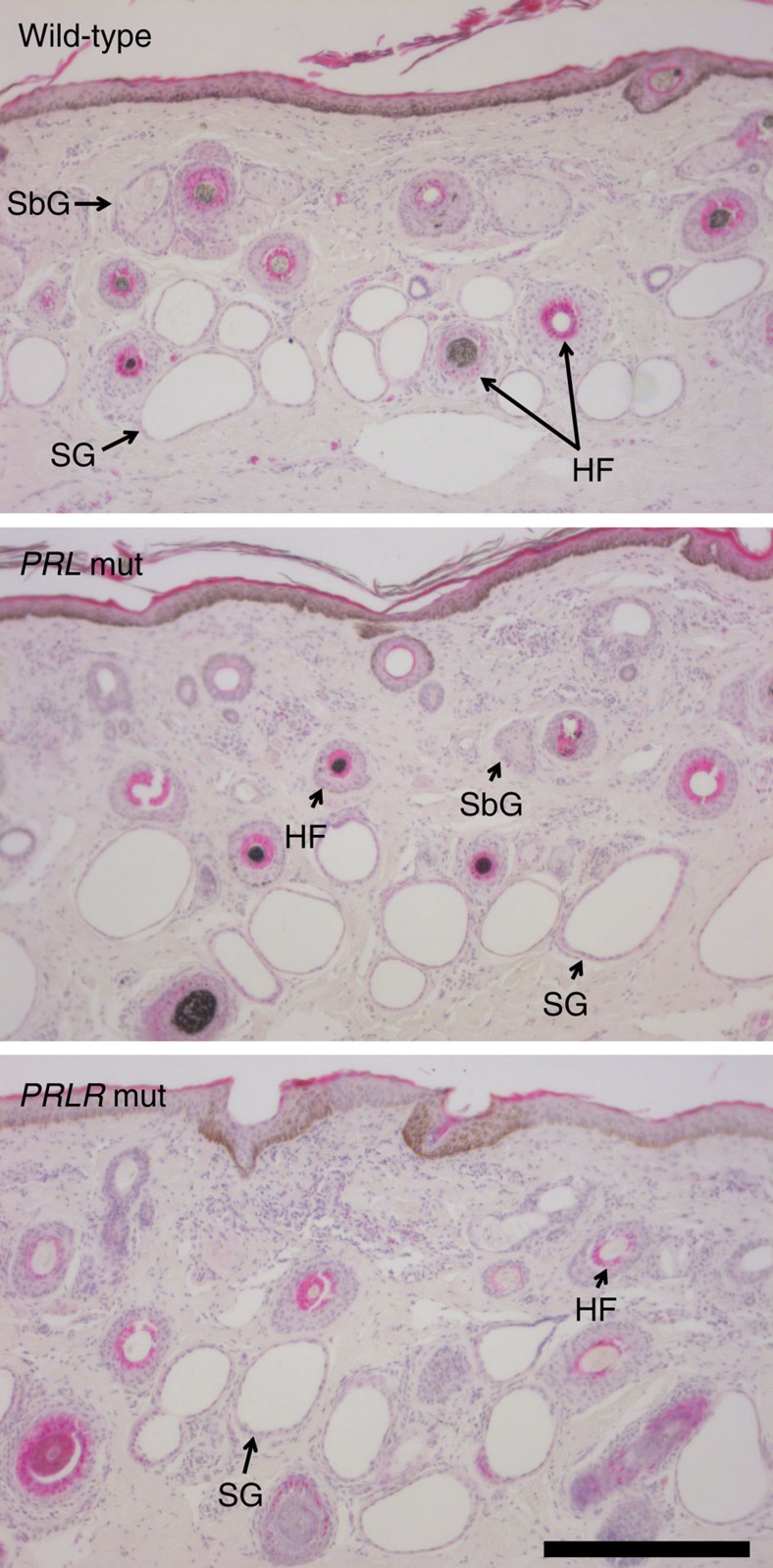
Skin histology of hairy and slick cattle. Example haemotoxylin/eosin-stained skin sections at 100 × magnification representing wild-type (*N*=11), hairy (*N*=12) and slick (*N*=3) cows. The epidermis is top of field in each panel, sweat glands (SG), sebaceous glands (SbG) and hair follicles with and without fibre cross-sections (HF) are indicated. No qualitative or quantitative differences were observed between the different genotypes. Scale bar: 300 μm.

Histological analyses of ear tissue biopsies were conducted using 12 wild-type, 11 *PRL* mutant and three *PRLR* mutant animals to further investigate the cutaneous phenotypes of hairy and slick cattle. Although the number of samples representing *PRLR* p.Leu462* carriers precluded formal statistical analysis, there appeared to be no differences in the size, shape and density of sweat glands or hair follicles compared with wild-type animals (Fig. 4). Notably, the sweat glands of *PRL* p.Cys221Gly mutants were indistinguishable from wild-type cows, despite the dysfunctional sweating exhibited by these animals. Likewise, there were no other qualitative or quantitative anatomical differences between wild-type and hairy syndrome skin sections (Fig. 4; Supplementary Table 6). This included hair follicle density, a result consistent with analysis of length and diameter-adjusted hair weight data.

**Figure 5:**
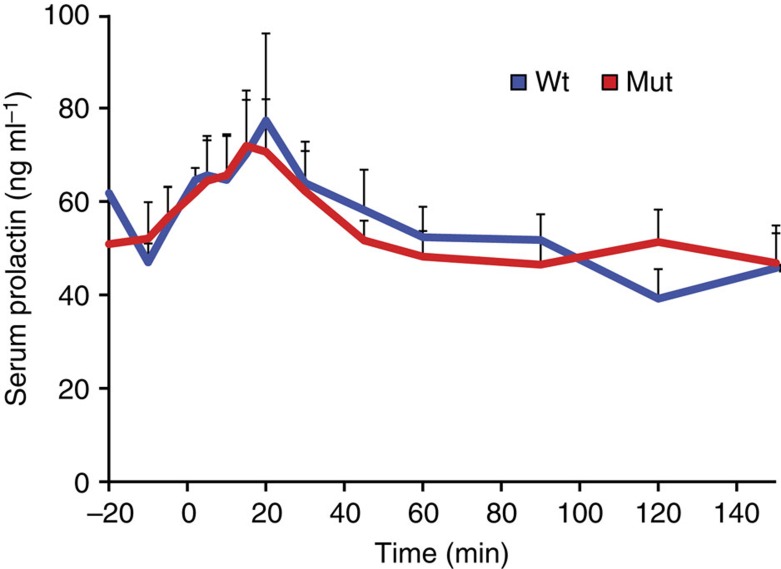
Prolactin secretory responses to TRH infusion. Serum ELISA results showing mean prolactin secretory responses to TRH challenge in *PRL* p.Cys221Gly mutant (*N*=6) and wild-type (*N*=6) animals. The *x* axis denotes time relative to TRH infusion (time=0), only positive values for error bars (s.e.m.) are plotted. Peak serum prolactin response was not significantly different between groups (two-sided *t*-test, *P*=0.96).

To investigate the molecular mechanism of prolactin dysfunction in hairy syndrome animals, we obtained pituitary samples representing four *PRL* mutant and four unrelated controls. Sequencing of pituitary RNA showed no difference in the expression of *PRL*, with western blotting of pituitary extracts also indicating comparable levels of prolactin peptide between groups (Supplementary Fig. 3). Serum enzyme-linked immunosorbent assay (ELISA) data from mutant (*N*=6) and wild-type (*N*=6) animals also indicated comparable prolactin-secretory responses when infused with thyrotropin-releasing hormone (Fig. 5). Together, these data suggested that mutant prolactin transcripts and hormone are expressed in the pituitary gland, and are actively secreted into circulation.

## Discussion

The complementarity of phenotypes, genetic association data and predicted functional impact of the *PRL* and *PRLR* mutations strongly suggests these as causal in determining the characteristics of hairy and slick cattle. Associations between circulating prolactin and thermal stress have been observed in various mammals including humans^18^, although a direct modulatory role for prolactin in thermoregulation has remained unproven. Our findings confirm such a role, presenting two bovine models to further explore these responses. Remarkably, these effects appear to occur through control of sweat secretion, with histological similarities between wild-type and *PRL* mutant cows indicating an acute signalling role for prolactin, as opposed to one affecting sweat gland morphogenesis.

These observations suggest that the *PRLR* p.Leu462* mutation may confer additional thermotolerance to cattle beyond its effects on short coat length. Two studies of Senepol–Holstein crossbreeds suggest that slick cattle sweat at higher rates than nonslick controls^19^,^20^. These studies present conflicting data regarding the mechanism of increased sweating rate, proposing this as a secondary effect related to coat length^19^, and alternatively as a consequence of genuinely higher secretory capacity^20^. The precise role of the *PRLR* mutation in sweating rate remains to be resolved; however, observations in hairy syndrome cattle would suggest direct secretory control. The reciprocity of *PRL* and *PRLR* mutations on coat length (and possibly sweating), and the observation of severe lactation dysfunction in hairy syndrome animals also suggests a role for the *PRLR* p.Leu462* mutation on lactation phenotypes. This seems especially likely, given the milk fat and protein yield effects attributed to a p.Ser18Asn substitution in bovine *PRLR*^21^. In studies where the *slick* haplotype has been introgressed into Holstein dairy cattle, slick-haired animals demonstrate higher milk yields than nonslick contemporaries^14^,^20^. These effects are assumed to be due to enhanced thermotolerance, with one study presenting winter milk yield data for which there was no apparent difference between slick and nonslick cows^20^. It should be noted however that the number of slick animals in that study was small (*N*=11), leaving the role of the *PRLR* p.Leu462* variant in lactation an open-ended question.

The molecular mechanisms by which the *PRL* and *PRLR* mutations could exert their effects remain unclear. Mutant and wild-type *PRL* transcripts are equivalently expressed in the pituitary gland, and the level of prolactin hormone is also similar between groups. Stimulated release of prolactin also appears comparable between hairy syndrome animals and controls. Although the relevance of these data to extrapituitary sites of prolactin synthesis is unknown, these findings suggest a receptor-binding-based mechanism underpinning the hairy syndrome, possibly involving receptor antagonism, given the haplosufficiency exhibited by *Prl* knockout mice^12^. The dominance of the *PRLR* mutation is also curious, since truncation of 120 C-terminal amino acids could be expected to result in a loss of function. Prolactin receptor knockout mice exhibit marginally larger diameter hair, although other phenotypes are reminiscent of hairy syndrome cattle, namely longer hair fibres, and failure to lactate^9^,^13^. These observations suggest enhanced prolactin pathway signalling as a result of the *PRLR* p.Leu462* mutation. An example of a functionally coupled, C-terminal *PRLR* mutant has recently been described in chickens, where, notably, this variant has been proposed as the causative mutation underlying a dominantly inherited feather-growth retardation phenotype^22^.

Irrespective of molecular mechanism, discovery of the *PRL* p.Cys221Gly and *PRLR* p.Leu462* mutations highlights new facets of prolactin biology, expanding the already-extensive repertoire of exocrine functions attributed to this hormone. The impact of the *PRLR* p.Leu462* mutation on thermotolerance carries additional industrial significance, and represents one of few dominant, beneficial alleles reported in livestock. This is of particular interest in dairy farming contexts, where most selection has occurred in heat-intolerant *Bos taurus* breeds. As a frameshift mutation, its amenability to gene editing will allow relatively simple assessment within diverse genetic backgrounds, potentially unlocking hot farming environments to the highest performance genetic lines.

## Methods

### Primary data

Genotype, phenotype and sequence data sets representing all experimental populations have been deposited in the Dryad digital data repository (http://doi.org/10.5061/dryad.nh6v4^23^), and NCBI SRA (SRP043521). Semen representing *PRL* p.Cys221Gly heterozygous animals may also be available for research purposes on request.

### Ethics statement

Ethics approval for all animal experiments was granted by the Ruakura Animal Ethics Committee, Hamilton, New Zealand, under approvals 13134 (heat-stress measurements), 13198 (thyrotropin-releasing hormone (TRH) infusion experiment) and 13346 (sweating analysis).

### Animal populations

Individuals used for genetic analyses of the hairy syndrome comprised a two-generation pedigree of 2,274 animals of predominantly Holstein–Friesian ancestry. This pedigree consisted of two large sire families representing the presumed *de novo* sire (67 progenies), and one of his affected male offspring (2,185 progenies). In addition, included were 21 dams forming 22 nuclear trios used for genome-wide analysis. Individuals targeted for *PRLR* genotyping and coat-length analysis consisted of four purebred Senepol sires, three Senepol × Charolais F1 sires, 41 crossbred animals of mixed Senepol, Barzona, Red Angus and Hereford ancestry, and 38 crossbred animals of predominantly Senepol, Red Angus and Tuli ancestry. Three Senepol × Holstein–Friesian animals were assessed for skin histological analysis. Genetic mapping was conducted retrospectively with sample sizes representing all animals for which phenotypic data were available. For prospective analyses (that is, heat stress, TRH infusion and sweating measurements), power calculations were conducted to restrict sample sizes to a minimum based on ethical approvals.

### Phenotypic analysis

Phenotypic classification across hairy and slick cohorts was made visually, the former coded as a binary trait and the latter on a quantitative coat-length scale scored 1–4. Slick cohort quantitative scores were also re-classified for binomial analysis into slick (score 1) and not slick (scores 2–4) classes. There were 37 cases and 62 controls representing 22 nuclear trios and 55 half-sibs used for mapping of the *hairy* locus. For *slick* analysis, the distribution of coat lengths is indicated in Supplementary Table 3.

Cows representing the hairy pedigree were distributed across various North Island and South Island commercial farms, with lactation data for affected (*N*=111) and unaffected half-sibs (*N*=760) extracted from a national database of milk yield and composition test results. Milk yield data were absent for 30% of affected animals compared with 3% of controls, largely due to failure of these animals to initiate lactation. Cows were tested at ~60 days of lactation, with >90% of all records measured during spring (September to November) in 2013. The mean daily temperatures reported in text represent the 3-month average for September to November 2013, with source temperature data obtained from the National Institute of Water and Atmospheric Research (https://www.niwa.co.nz/).

For quantitative assessment of hair phenotypes, a 100-cm^2^ area of skin was clipped in the left dorsolumbar region of 12 hairy and 12 control cows (Supplementary Fig. 1). Collected hair was weighed with a subsample photographed on a glass microscope slide. Images were analysed using ImageJ (http://imagej.nih.gov/ij/), with randomly selected hairs measured for diameter (*N*=20) and length (*N*=10).

Measurements of rectal temperature, respiration rate and heart rate were made on the same 24 cows used for hair morphological analysis, assessed outdoors without shade in the morning (9a.m.) and afternoon (3p.m.) on two consecutive days. Rectal temperature was measured using a clinical thermometer. Respiration and heart rate were assessed over a 30-s period. Five of the twelve hairy cows were subsequently clipped with grooming shears to a coat-length-matching control cows (Supplementary Fig. 1). Respiration rate and rectal temperature were then measured 5 days later to allow for re-acclimation.

Measurement of sweating rates was conducted indoors in a heated room maintained at 28 °C. Six affected and six age-matched control animals (two unaffected half-sibs and four unrelated animals) were used for analysis, assessed in batches of four animals per measurement period. Sweating rates (g m^−2^ skin area per h) were measured by the CaCl_2_ capsule method^24^, using inverted 82-mm diameter Petri dishes filled with 50 g anhydrous CaCl_2_ (Sigma-Aldrich), separated from the skin by a gauze membrane. Animals were introduced to the hot room 1 h before sweat measurement, with capsule weight change measured over the following hour. The mean sweating rate across two clipped skin areas per animal was quantified, measured at the fore flank posterior the right shoulder and the right dorsolumbar region. Respiration rates and rectal temperatures were also measured at the end of the heat exposure period (Supplementary Fig. 2).

### Sanger sequencing and custom genotyping

Semen, hair or ear punch tissue samples were used for DNA extraction following standard protocols, with samples processed by GeneMark (Hamilton, New Zealand) or GeneSeek (Lincoln, NE, USA). For Sanger sequencing of *PRL*, primers were designed to amplify all exons, intron–exon boundaries, and 2 kb of 5′ non-coding sequence according to the RefSeq transcript NM_173953 (Supplementary Table 7). For *PRLR* sequencing, all exons, intron–exon boundaries and 3 kb of 5′ non-coding sequence were amplified according to annotations derived from mammary RNA-sequence data (not shown), targeting an additional 5′ untranslated region (UTR) exon and 9 kb of additional 3′ UTR sequence relative to the RefSeq gene structure NM_001039726 (Supplementary Table 7). Amplicons were sequenced using BigDye version 3.1 chemistry on a 3130xL instrument (Applied Biosystems) at the University of Auckland DNA Sequencing Facility (Auckland, New Zealand). Custom genotyping of the chr23:35105313A>C *PRL* SNP was performed by GeneMark using a TaqMan assay (Applied Biosystems). Genotyping of the chr20:39136558GC>G *PRLR* variant was conducted by GeneSeek using Sequenom iPLEX (Sequenom), targeting alleles in both forward and reverse strand orientations.

### High-throughput genotyping and imputation

For genome-wide analysis within the hairy pedigree, 74 animals were genotyped using the Illumina BovineSNP50 BeadChip (Illumina), and 24 using the GeneSeek Genomic Profiler BeadChip (Super GGP; GeneSeek/Illumina). These data were used to impute a total of 712,123 SNPs from the Illumina BovineHD BeadChip using Beagle software^25^ (v4), from a reference population of 3,222 animals. Senepol and Senepol crossbred animals were typed using the BovineSNP50 BeadChip. All variant positions reference the UMD3.1 *Bos taurus* genome assembly.

### Association analysis

Milk yield phenotypes were derived from linear models fitted to a wider data set that included all herd contemporaries. Residuals from these models, which included milk yield as the dependent variable, and independent variables for herd, stage of lactation, age at calving, breed and heterosis were used for association testing based on two-sided *t*-tests. Cows were also stratified for analysis based on their geographical location (North or South Island). Two-sided *t*-tests were also used to evaluate associations with heat tolerance traits, hair-related traits and histological and molecular phenotypes.

All genotype data were filtered to exclude markers for minor allele frequency (<1%), and per-individual genotype call rate (<90%). Family-based genome-wide association testing in the hairy pedigree was conducted using the DFAM procedure in PLINK^26^, combining both full and incompletely genotyped trios in a single TDT-based analysis. For haplotype analysis of the slick locus, the Beagle software was used to phase the *PRLR* p.Leu462* mutation together with 25 Illumina SNP50 BeadChip SNPs representing the chromosome 20 38.6–39.6 Mbp target interval. Six-marker sliding window haplotypes were used to span the interval, incorporating a three-marker overlap per tile (50% redundancy). Individual haplotypes (minimum *N*=5) were tested for association with coat length using two-sided *t*-tests, assuming a dominance model. For association analysis of the *PRLR* genotype with coat length, dominant genetic models were assessed using genotypic tests in PLINK. An alpha level of 0.05 was used for all tests, incorporating Bonferroni corrections for multiple hypothesis testing within each experiment. Associations were considered significant at *P*<0.016 for lactation, hair morphology, sweating rate and histological analyses (three tests each), and *P*<0.002 for heat stress phenotypes (24 tests). Associations from DFAM analysis were considered significant at *P*<7.96 × 10^−8^ (628,278 tests), haplotype tests were considered significant at *P*<7.14 × 10^−4^ (70 tests). Unadjusted *P* values are reported in text.

### Genome and exome sequencing

Whole-genome 100-bp paired-end sequencing was performed by Illumina FastTrack using the Illumina HiSeq 2000 instrument. Sequenced animals included 135 Holstein–Friesians, 102 Jerseys, 316 Holstein–Friesian × Jersey crossbreeds and 1 Ayrshire. The mean mapped read-depth was 11 × per animal with a median mapped depth of 7 × . The sequence database included the unaffected father (6 × mapped depth) of the presumed *de novo* sire, with the two *PRL* mutant sires sequenced to a mapped depth of 8 × . Exome sequencing was conducted on 115 animals representing Holstein–Friesian (*N*=10), Jersey (*N*=10), Angus (*N*=9), Belgian Blue (*N*=29), Brahman (*N*=10), Charolais (*N*=10), Nelore (*N*=10), Senepol (*N*=9), Simmental (*N*=10) and Yak (*N*=8) breeds. Custom capture targeting all RefSeq, Ensembl and human paralogous genes was performed using the SureSelect Target Enrichment System (Agilent), with 101-bp paired-end sequencing conducted on the HiSeq 2000. The mean sequencing depth across exome targets was 25–40 × per sample.

### Sequence informatics and variant filtering

Sequence reads were aligned to the *Bos taurus* UMD3.1 genome assembly using RTG map^27^ (v2.7.2) for genome sequence, and BWA aln^28^ (v0.6.2) for exome data. Variant calling was performed using RTG Population^27^ caller and GATK HaplotypeCaller^29^ (v2.8) on genome and exome alignments, respectively. Variant lists were filtered based on affection status criteria and functional predictions according to gene structures from Ensembl gene build 74.

### Histological analysis

Skin samples were obtained by biopsy using a 3.5-mm needle ear-punch (Allflex, Brisbane, Australia). Eleven hairy syndrome and twelve wild-type animals were sampled for analysis, with 18 of these overlapping with the animals used for hair morphological analysis, and the remainder representing unrelated wild-type animals. Three Senepol x Holstein–Friesian crossbreeds heterozygous for the *PRLR* p.Leu462* mutation were also sampled for analysis. Tissue samples were fixed for 24 h in 10% neutral-buffered formalin, dehydrated and embedded in paraffin wax. Tissues were then sectioned perpendicular to the skin surface at 7 μm and stained with haemotoxylin and eosin. Slides were visualized using a DMI 3000 B research microscope (Leica) and images captured using a DFC295 camera (Leica). For quantitative assessment of sweat gland and hair follicle density phenotypes, skin surface length was measured using ImageJ software, with features counted and normalized to this length. As a proxy of sweat gland size, sweat gland perimeters were also measured, with the mean perimeter length per individual used for statistical analysis. Since biopsies were double-sided (that is, represented both ‘inside’ and ‘outside’ ear surfaces), both surfaces were used for quantification.

### Pituitary RNA sequencing and western blotting

Pituitaries were obtained from four hairy syndrome animals and four unrelated age-matched control calves following their killing on commercial slaughter premises. Pituitaries were pulverized in liquid nitrogen with samples divided for protein and RNA analyses. For RNA extraction and sequencing, tissue was homogenized in TRIzol Reagent (Life Technologies) and total RNA recovered using standard protocols by NZ Genomics Limited (NZGL; Auckland, NZ). Illumina sequencing libraries were prepared and sequenced by NZGL (Dunedin, NZ) using 100-bp paired-end reads on the HiSeq 2000 instrument, yielding 24–30 million read pairs per sample. Reads were mapped with Tophat2 (ref. 30) (v2.0.8), and *PRL* expression quantified using the ‘variance stabilizing transformation’ function in DESeq^31^ (v1.14.0). Pituitary protein extracts were resolved on 12% SDS–PAGE gels and blotted on polyvinylidene difluoride (Bio-Rad). Western blotting was performed using antibodies to bovine prolactin (1:1,000 National Hormone and Peptide Program, USA) with beta-tubulin included as a loading control (1:2,000 AbCam ab6046).

### TRH challenge

TRH infusion was conducted using the same 12 animals used for analysis of sweating rate. Jugular catheters were inserted on the day before the challenge, with catheter patency maintained with heparinized saline. Lyophilized hormone was obtained from Peptide Sciences (http://www.peptidesciences.com/trh) as 99% pure. Peptide was reconstituted in PBS at 20 μg ml^−1^ and administered at 0.3 μg TRH per kg of body weight. Blood samples (10 ml) were collected at −20, −10, −5, 2, 5, 10, 15, 20, 30, 45, 60, 90, 120 and 150 min relative to the time of the TRH bolus injection. Blood plasma was separated using centrifugation and ELISA assays conducted with AgResearch (Ruakura Research Centre, Hamilton), using bovine prolactin kits (USCN Life Science Inc., Cat. No. CEA846Bo).

## Author contributions

M.D.L., K.M.H., R.J.S. and S.R.D. conceived, designed and interpreted the experiments; K.M.H. conducted functional experiments; T.J., C.H., T.L. and W.L. conducted sequence analysis; M.D.L., K.T. and R.G. Sherlock performed statistical analyses; S.D.L., B.C.S. and D.J.G. provided animal resources; M.D.L., R.G. Snell, R.J.S. and S.R.D. supervised the project; M.D.L. wrote the manuscript.

## Additional information

**How to cite this article:** Littlejohn, M. D. *et al*. Functionally reciprocal mutations of the prolactin signalling pathway define hairy and slick cattle. *Nat. Commun.* 5:5861 doi: 10.1038/ncomms6861 (2014).

**Accession codes:** Genotype, phenotype and sequence data sets representing all experimental populations have been deposited in NCBI Short Read Archive under accession code SRP043521 and in Dryad Digital Repository under DOI: 10.5061/dryad.nh6v4.

## Supplementary Material

Supplementary Information Supplementary Figures 1-3 and Supplementary Tables 1-7
