# Supplementary material for: Functionally reciprocal mutations of the prolactin signalling pathway define hairy and slick cattle
Source: Nat Commun. 2014 Dec 18;5:5861. doi: 10.1038/ncomms6861 (PMC4284646; doi:10.1038/ncomms6861)
Supplement: Supplementary Information — Supplementary Figures 1-3 and Supplementary Tables 1-7 [file ncomms6861-s1.pdf]

Supplementary Information

Functionally Reciprocal Mutations of the Prolactin Signalling Pathway Define Hairy and Slick Cattle

Mathew D Littlejohn[1,2]*, Kristen M Henty[2]*, Kathryn Tiplady[1], Thomas Johnson[1], Chad Harland[1], Thomas Lopdell[1], Richard G Sherlock[1], Wanbo Li[3], Steven D Lukefahr[4], Bruce C Shanks[5], Dorian J Garrick[6], Russell G Snell[2], Richard J Spelman[1] & Stephen R Davis[1]

1. Livestock Improvement Corporation, Cnr Ruakura & Morrinsville Roads, Newstead, Hamilton 3240, New Zealand
2. School of Biological Sciences, University of Auckland, 3A Symonds Street, Auckland 1010, New Zealand
3. Unit of Animal Genomics, GIGA-R & Faculty of Veterinary Medicine, University of Liège (B34), Liège, Belgium
4. Department of Animal, Rangeland & Wildlife Sciences, MSC 228, Texas A&M University-Kingsville, Texas 78363-8202, USA
5. Department of Agriculture and Environmental Science, Lincoln University, 820 Chestnut Street, Jefferson City, Missouri 65101, USA
6. Department of Animal Science, Iowa State University, 225 Kildee, Ames, Iowa 50011-3250, USA

* These authors contributed equally to this work

Contents

Supplementary Figure 1 2

Supplementary Figure 2 3

Supplementary Figure 3 5

Supplementary Table 1 6

Supplementary Table 2 8

Supplementary Table 3 9

Supplementary Table 4 10

Supplementary Table 5 13

Supplementary Table 6 14

Supplementray Table 7 15

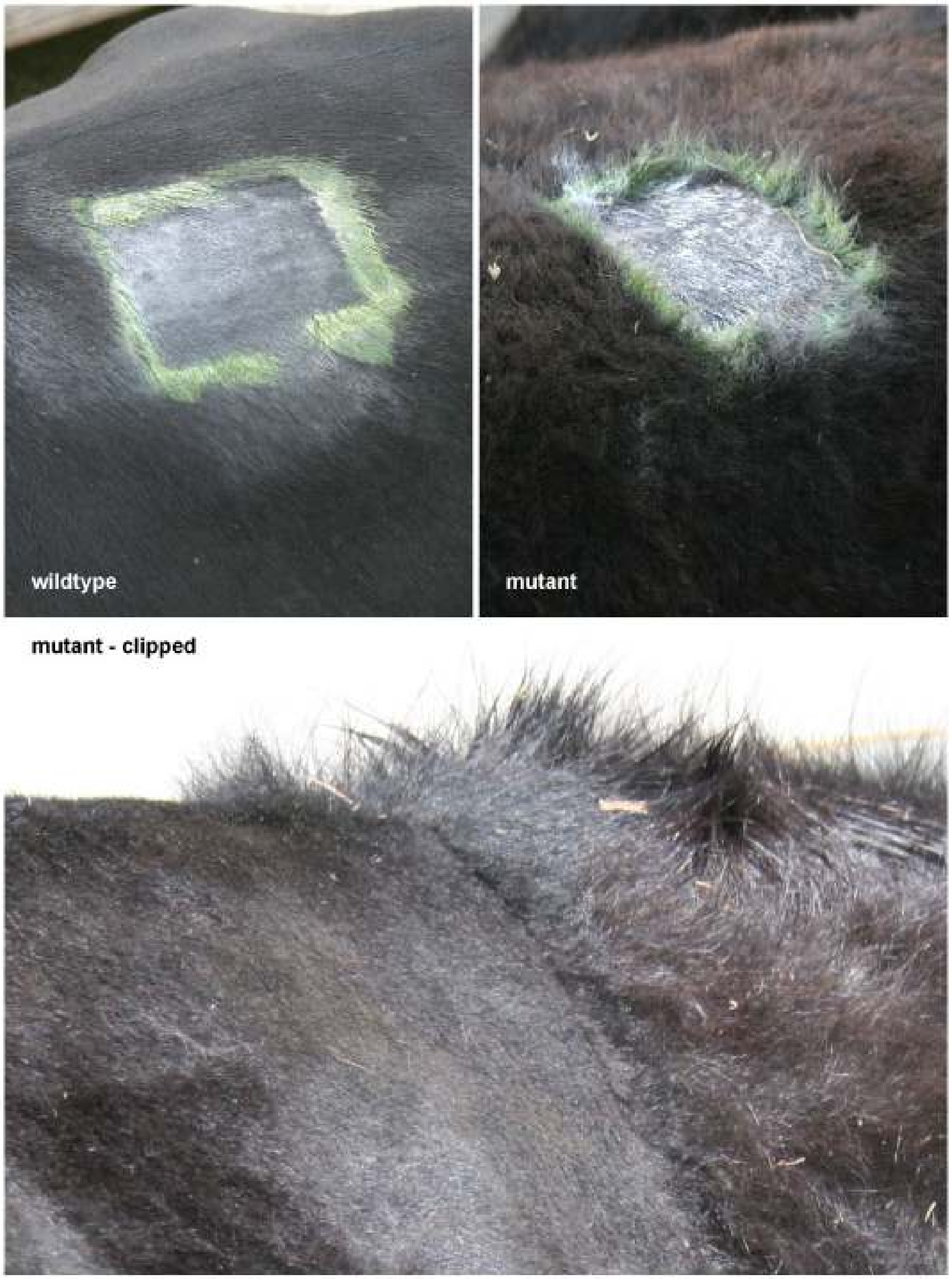

Supplementary Figure 1: Coat analysis of hairy syndrome cattle

Photographs indicating coat differences between wild-type and mutant half-sibs (top), showing shaved skin areas used for collection of hair for morphological analysis (the white appearance is due to application of sunscreen). The bottom photograph shows contrast of coat-length in a clipped mutant animal, where coat-length was reduced to match those of wild-type animals for heat stress response measurements.

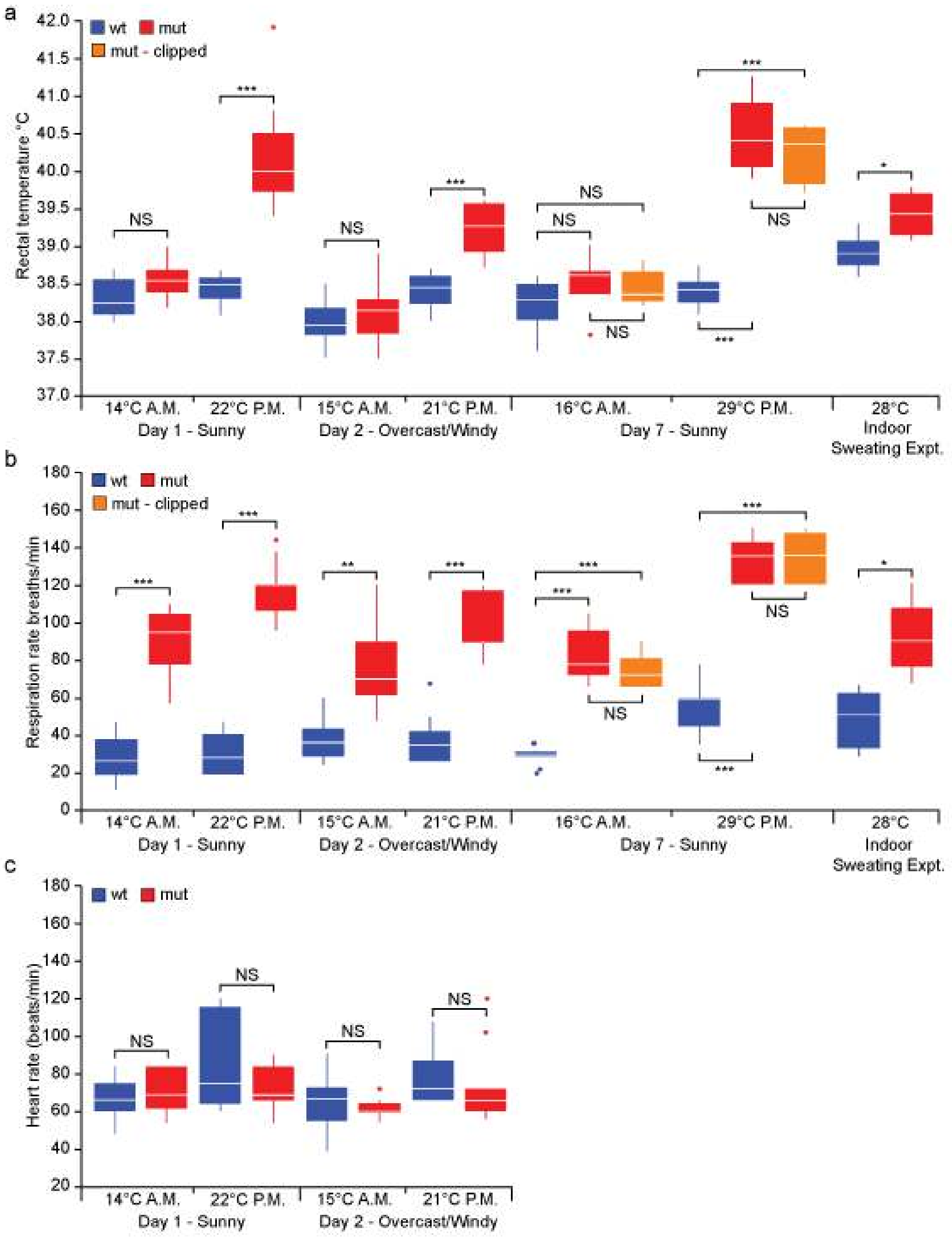

Supplementary Figure 2: Heat stress response measures of hairy pedigree animals at varying ambient temperatures

Rectal temperature (**a**), respiration rate (**b**), and heart rate (**c**) responses to different ambient temperatures in mutant and wild-type half-sibs. These data include additional time-points to those displayed in Figure 1, representing morning and afternoon measurements made on four separate days. Day 1 and Day 2 measurements compare 12 wild-type and 12 mutant animals, Day 3 measurements contrast the responses of clipped mutant animals (N=5) to un-clipped mutant (N=7), and control (N=12) animals. Rectal temperature and respiration rate data for the indoor sweating rate experiment are also indicated. Box plots define the median, upper and lower quartiles for the various phenotypes, with whiskers representing the furthest data-points within 1.5x of the interquartile range, and outlier samples indicated beyond this range. *P<0.05, **P<0.001, ***P<0.0001 (two-sided *t* tests, Bonferroni-adjusted).

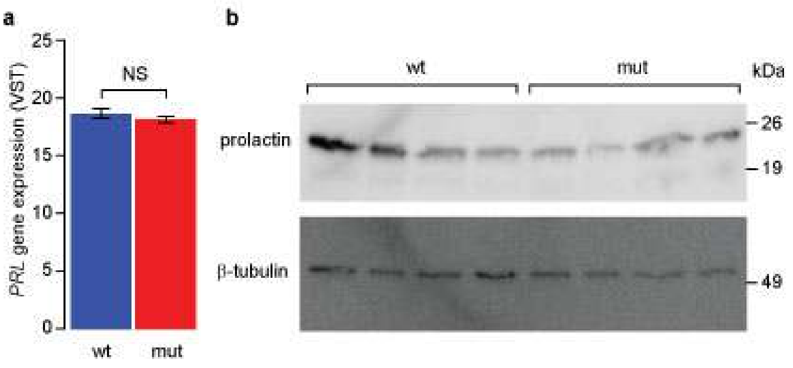

Supplementary Figure 3: Pituitary prolactin gene and protein expression

(**a**) Mean pituitary prolactin gene expression in *PRL* mutant (N=4) and control (N=4) animals, quantified using RNA sequencing. Data represent variance transformed read counts, with no significant difference observed between groups (two-sided *t* test, P=0.32; error bars are ±s.e.m). Allele-tagged read counts within mutant samples was also similar for mutant and reference alleles (data not shown; between 51-54% 'C'-tagged reads for each of the four heterozygous mutant animals). (**b**) Prolactin western blot of pituitary extracts representing the same samples used for RNA sequencing, showing comparable levels of pituitary hormone between mutant and wild-type animals.

Phenotype	Control N	Control Mean	Control SE	Mutant N	Mutant Mean	Mutant SE	Clipped Mutant N	Clipped Mutant Mean	Clipped Mutant SE	*t* value	*P*-value	Bonferroni *P*
Milk Yield (L)												
Milk Yield – NZ-wide	740	18.9	0.14	77	14.3	0.57	.	.	.	-9.71	3.73E-21	1.12E-20
Milk Yield – North Island	446	18.7	0.17	39	14.9	0.78	.	.	.	-6.23	1.00E-09	3.00E-09
Milk Yield – South Island	294	19.1	0.24	38	13.8	0.84	.	.	.	-7.38	1.27E-12	3.81E-12
Hair Morphology												
Hair length (mm)	12	11.6	0.65	12	26.8	1.9	.	.	.	-7.58	1.42E-07	4.26E-07
Hair diameter (microns)	12	67.2	3.72	12	46.4	2.62	.	.	.	4.58	1.48E-04	4.44E-04
Hair weight (mg/cm2 skin)	12	9.1	0.68	12	16.1	2.46	.	.	.	-2.73	0.012	0.037
Heat stress												
Rectal Temperature (°C)												
Day 1 A.M. 14°C	12	38.3	0.068	12	38.6	0.073	.	.	.	-2.5	0.021	0.504
Day 1 P.M. 22°C	12	38.5	0.053	12	40.2	0.193	.	.	.	-8.73	1.33E-08	3.19E-07
Day 2 A.M. 15°C	12	38	0.085	12	38.1	0.116	.	.	.	-0.93	0.364	1
Day 2 P.M. 21°C	12	38.4	0.063	12	39.2	0.094	.	.	.	-6.91	6.19E-07	1.49E-05
Day 3 A.M. 16°C	12	38.2	0.083	7	38.5	0.138	.	.	.	-1.73	0.101	1
Day 3 A.M. 16°C	12	38.2	0.083	.	.	.	5	38.4	0.104	-1.32	0.280	1
Day 3 A.M. 16°C	.	.	.	7	38.5	0.138	5	38.4	0.104	0.37	0.716	1
Day 3 P.M. 29°C	12	38.4	0.054	7	40.5	0.187	.	.	.	-13.25	2.17E-10	5.21E-09
Day 3 P.M. 29°C	12	38.4	0.054	.	.	.	5	40.2	0.175	-13.23	1.13E-09	2.71E-08
Day 3 P.M. 29°C	.	.	.	7	40.5	0.187	5	40.2	0.175	1.01	0.337	1
Indoors 28°C (Sweating Expt)	6	38.9	0.098	6	39.5	0.11	.	.	.	-3.59	0.005	0.015
Respiration Rate (breaths/min)												
Day 1 A.M. 14°C	12	29	3.2	12	91	5	.	.	.	-10.49	5.04E-10	1.21E-08
Day 1 P.M. 22°C	12	32	3.1	12	118	3.9	.	.	.	-17.34	2.57E-14	6.17E-13
Day 2 A.M. 15°C	12	37	2.9	12	75	5.8	.	.	.	-5.87	6.62E-06	1.59E-04
Day 2 P.M. 21°C	12	35	3.6	12	99	4.3	.	.	.	-11.45	9.73E-11	2.34E-09
Day 3 A.M. 16°C	12	30	1.4	7	81	5.4	.	.	.	-11.69	1.50E-09	3.60E-08

Day 3 A.M. 16°C	12	30	1.4	.	.	.	5	73	4.4	-12.61	2.20E-09	5.28E-08
Day 3 A.M. 16°C	.	.	.	7	81	5.4	5	73	4.4	1.05	0.316	1
Day 3 P.M. 29°C	12	57	3.3	7	132	4.6	.	.	.	-13.52	1.59E-10	3.82E-09
Day 3 P.M. 29°C	12	57	3.3	.	.	.	5	134	6.1	-11.98	4.40E-09	1.06E-07
Day 3 P.M. 29°C	.	.	.	7	132	4.6	5	134	6.1	-0.26	0.800	1
Indoors 28°C (Sweating Expt)	6	48	6.2	6	91	7.7	.	.	.	-4.33	0.002	0.006
Heart Rate (beats/min)												
Day 1 A.M. 14°C	12	67	3.1	12	71	3.4	.	.	.	-0.98	0.336	1
Day 1 P.M. 22°C	12	85	7	12	74	3.2	.	.	.	1.49	0.149	1
Day 2 A.M. 15°C	11	65	4	12	62	1.3	.	.	.	0.75	0.462	1
Day 2 P.M. 21°C	12	77	3.8	12	73	5.5	.	.	.	0.67	0.507	1
Sweating Rate (grams/m^2 skin area/hr)												
Indoors 28°C	6	225	40	6	111	49	.	.	.	4.45	0.001	0.004

Supplementary Table 1: Association statistics for lactation, hair morphology, and heat stress phenotypes for hairy syndrome mutant and wild-type half-sibs

CHR	POS	HOMREF	HET	HOMALT	EFFECT	Amino_Acid_Change	Gene_Name	Transcript_ID
Chr23	27549182	554	2	0	DOWNSTREAM		NFKBIL1	ENSBTAT00000035817
Chr23	27549182	554	2	0	DOWNSTREAM		NFKBIL1	ENSBTAT00000019268
Chr23	27549182	554	2	0	INTERGENIC			
Chr23	27610230	554	2	0	INTERGENIC			
Chr23	27610230	554	2	0	UPSTREAM		ENSBTAG00000031913	ENSBTAT00000055663
Chr23	30971021	554	2	0	INTRON		ZNF184	ENSBTAT00000015283
Chr23	35105313	554	2	0	NON_SYNONYMOUS_CODING	C221G	PRL	ENSBTAT00000020313
Chr23	35159091	554	2	0	INTERGENIC			
Chr23	39362478	554	2	0	INTERGENIC			
Chr23	47446572	554	2	0	INTRON		BMP6	ENSBTAT00000025614

Supplementary Table 2: Annotation details for the seven chromosome 23 variants identified from whole genome sequence analysis of the two hairy syndrome founder sires

	PRLR p.Leu462* carrier	
Hair Length	Yes	No
1	41	1
2	2	21
3	0	11
4	0	6
Slick		
Yes	41	1
No	2	38

Supplementary Table 3: Distribution of quantitative and binary hair-length scores for *PRLR* p.Leu462* carrier and wild-type crossbreeds

Tile	Chr20 Position (bp)	Tile Size (kb)	Haplotype	Haplo Count by Coat-Length 1	2	3	4	Total	*P*-value	Bonferroni *P*
1.1	38606353-38892683	286	CCAAGT	6	4	2	1	13	0.749	1
1.2			CTAATT	3	7	1	3	14	0.293	1
1.3			TCGCGT	1	3	2	0	6	0.287	1
1.4			TCGCTT	46	6	5	4	61	4.00E-05	0.003
1.5			TTAAGT	7	0	0	2	9	0.735	1
1.6			TTGAGT	8	21	4	0	33	0.484	1
1.7			TTGCGT	10	1	2	2	15	0.875	1
2.1	38794140-38989299	195	AGTAAA	6	0	0	2	8	0.955	1
2.2			AGTAAG	5	2	2	1	10	0.642	1
2.3			AGTAGG	11	18	3	0	32	0.818	1
2.4			AGTGGG	0	5	1	0	6	0.287	1
2.5			ATTGGG	3	10	1	3	17	0.313	1
2.6			CGTAGG	8	3	5	2	18	0.146	1
2.7			CTTAGG	42	2	2	0	46	1.23E-16	8.60E-15
2.8			CTTGAG	3	3	5	4	15	1.95E-04	0.014
3.1	38920878-39071965	151	AAAGCC	6	0	0	2	8	0.955	1
3.2			AAGACT	6	3	3	1	13	0.524	1
3.3			AGGACC	53	7	3	2	65	1.42E-06	9.97E-05
3.4			AGGACT	4	7	3	0	14	0.490	1
3.5			AGGGCC	5	8	3	0	16	0.743	1
3.6			AGGGCT	1	2	2	0	5	0.296	1
3.7			GAGACT	3	3	5	4	15	0.000195	0.014
3.8			GGGACC	1	8	1	0	10	0.412	1
3.9			GGGGCC	2	8	1	3	14	0.163	1
4.1	39017985-39141815	124	ACCG*T	43	2	0	0	45	2.40E-19	1.68E-17
4.2			ACCGCT	10	1	1	2	14	0.589	1
4.3			ACCTCT	4	12	3	0	19	0.822	1

4.4			ACTTCC	10	11	9	5	35	0.007	0.491
4.5			ACTTCT	3	2	2	0	7	0.797	1
4.6			GCCGCT	5	16	2	1	24	0.351	1
4.7			GCCTCT	8	0	2	4	14	0.329	1
4.8			GCTGCT	1	2	2	0	5	0.296	1
5.1	39100174-39242226	142	G*TGGA	42	2	0	0	44	8.39E-18	5.87E-16
5.2			GCTGGA	1	2	2	0	5	0.296	1
5.3			GCTGGG	3	7	0	0	10	0.809	1
5.4			GCTTAA	11	10	3	3	27	0.294	1
5.5			TCCTAA	9	8	6	1	24	0.588	1
5.6			TCCTAG	1	3	3	4	11	5.81E-06	4.07E-04
5.7			TCTGGA	3	8	5	0	16	0.369	1
5.8			TCTTAA	7	0	0	2	9	0.735	1
5.9			TCTTAG	4	6	0	2	12	0.362	1
6.1	39194261-39350230	156	GGACAC	41	2	0	0	43	1.89E-16	1.32E-14
6.2			GGATAT	3	1	2	0	6	0.863	1
6.3			GGATGT	1	8	5	0	14	0.100	1
6.4			GGGTAC	3	7	0	0	10	0.809	1
6.5			TAACAC	7	0	2	1	10	0.809	1
6.6			TAACAT	20	18	7	5	50	0.117	1
6.7			TAGTAC	4	9	3	4	20	0.001	0.085
7.1	39275917-39509383	234	CACTCG	21	0	0	0	21	5.28E-06	3.70E-04
7.2			CACTCT	5	0	2	1	8	0.739	1
7.3			CACTTT	22	2	0	0	24	3.17E-05	0.002
7.4			CATCCG	3	7	3	0	13	0.339	1
7.5			CATCTT	9	1	2	2	14	0.940	1
7.6			CATTCG	4	9	2	3	18	0.210	1
7.7			CATTCT	6	1	0	2	9	0.975	1
7.8			TACCCT	4	7	0	0	11	0.622	1
7.9			TACCTT	0	4	1	1	6	0.049	1

7.10			TACTCG	2	1	1	3	7	0.005	0.350
7.11			TATTTT	3	1	3	0	7	0.502	1
7.12			TGTCCT	1	6	1	0	8	0.470	1
7.13			TGTTTT	1	2	2	0	5	0.296	1
8.1	39422828-39576637	154	CCGCG	3	7	3	0	13	0.339	1
8.2			CCTCG	6	13	1	0	20	0.829	1
8.3			CTTCA	0	4	1	1	6	0.049	1
8.4			CTTCG	9	1	2	2	14	0.940	1
8.5			TCGCA	24	9	1	3	37	0.009	0.626
8.6			TCGTA	2	0	1	3	6	0.004	0.252
8.7			TCTCG	12	3	3	3	21	0.621	1
8.8			TTTCG	7	1	0	0	8	0.042	1
8.9			TTTTA	20	7	5	0	32	0.212	1

Supplementary Table 4: Association results for 6-marker sliding window haplotypes in 82 Senepol crossbreeds

Eight consecutive tiles (3-marker overlaps) cover the 38.6-39.6Mbp *slick* locus, representing 70 haplotype states. Haplotype sequences and distribution across coat-length scores are indicated, with the *PRLR* p.Leu462* mutation represented by a red asterisk. Peak significance is observed for tile 4.1, carrying the *PRLR* p.Leu462* mutation and nested within a larger, 229kb contiguous haplotype shared by 41 of 42 slick-coded animals (Supplementary Table 5). Notably, the ancestral-allele haplotype 4.2 is un-associated with coat length.

ID	Haplotype	N
1	GACCG*TGG	45
2	**GACTTCCTA**	35
3	**GGCCGCTTA**	14
4	GACCGCTTA	14
5	GGCCGCTGG	10
6	GACCTCTTA	9
7	GACCTCTGG	9
8	AGCCTCTTA	8
9	GACTTCTGG	7
10	GGCTGCTGG	5
11	GGCCTCTTA	4
12	GGCCTCTGA	2
13	GATCGCTTA	1
14	GACCTCTTG	1

Supplementary Table 5: Senepol crossbreed haplotypes spanning a 229kb interval at the *slick* locus

A 229kb interval on chromosome 20 (38989299-39218755bp) demarcating a contiguous haplotype block found in all *PRLR* p.Leu462* carriers, and 41 of 42 slick-coded animals (haplotype 1). The single slick-coded animal that did not carry the p.Leu462* mutation is heterozygous for haplotypes 2 and 3 (bolded).

Phenotype	Control N	Control Mean	Control SE	Mutant N	Mutant Mean	Mutant SE	*t* value	*P*-value	Bonferroni *P*
Histology									
Sweat gland density (glands/mm skin surface length)	12	1.454	0.157	11	1.255	0.164	0.88	0.390	1
Hair follicle density (follicles/mm skin surface length)	12	2.532	0.391	11	3.468	0.408	-1.65	0.113	0.339
Sweat gland perimeter length (mm)	12	0.472	0.034	11	0.447	0.035	0.51	0.616	1
Prolactin expression									
PRL gene expression (VST counts)	4	18.7	0.39	4	18.2	0.24	1.08	0.321	.
Peak serum prolactin (ng/mL)	6	157.5	12.93	6	158.7	20.16	-0.05	0.962	.

Supplementary Table 6: Association statistics for histological and prolactin expression phenotypes

	Primer Name	Sequence
PRL		
Promoter	PRL_Promo_For1	TTGGAGAAGGAAATGGCAAC
	PRL_Promo_Rev1	CGTCAACTTAAAGCTGGGTCA
	PRL_Promo_For2	TGGGGCAGCATTAATTTCTT
	PRL_Promo_Rev2	TCAGGAGGGATGTGAAGAGG
	PRL_Promo_For3	GTGTGCCCTTGAAAACCACT
	PRL_Promo_Rev3	CCAGAAATGAACATCTAGGAAGG
Exon 1	PRL_Exon_1_For1	TGCAGAGAAATAAAGGCAAATG
	PRL_Exon_1_Rev1	CAAATGTCTCTGGAAGACAGTCC
Exon 2	PRL_Exon_2_For1	TTTACACAGTGGAAGGTGTTGC
	PRL_Exon_2_Rev1	CAGGTGCTTTAAATTTATTTTGGAA
Exon 3	PRL_Exon_3_For1	GGATGAAATGAAACAAGGGAAA
	PRL_Exon_3_Rev1	CACCTTTCTCTGCATGTCCA
Exon 4	PRL_Exon_4_For1	GGTCAATCACTCTGAGCAAAAA
	PRL_Exon_4_Rev1	GCCATCTGTACCCAGGAAGA
Exon 5	PRL_Exon_5_For1	TGGCTCCAAAATCCAAGTGT
	PRL_Exon_5_Rev1	CCCAGAATAATTTGCTGTGATTC
3' UTR	PRL_3'_For1	CAACAACTGCTAAGCCCACA
	PRL_3'_Rev1	CCACCTGACCATTTCCAAAC
PRLR		
Promoter	PRLR_Promo_For1	GCAAATGGGATTCTCCAGAC
	PRLR_Promo_Rev2	GGAAAGAACCCAGCTTTTTG
	PRLR_Promo_For3	TGAGGTTTAGGGAAGCCAAG
	PRLR_Promo_Rev3	TTCCCTTTTGAATGATTGACAC
	PRLR_Promo_For4	GGACTCAGCAGGTGGCTACT
	PRLR_Promo_Rev5	GGTGTCCCAACCTGGACTC
Exon 1	PRLR_5UTR_For1	CCTCCTCTCGCAAAGAAAGA
	PRLR_5UTR_Rev2	TTAGGGTAAGGTGGGCTGCT
Exon 2	PRLR_Exon2_For2	TCGATACCTGGGTCTGGAAG
	PRLR_Exon2_Rev2	AACAGCAGAATGCAACAACG
Exon 3	PRLR_Exon3_For	ATTTTTCCCAGCGTATGCAC
	PRLR_Exon3_Rev	TCCCAGAATGAGGATGGAAG
Exon 4	PRLR_Exon4_For	TGCTGACATCTTGGCACTTC
	PRLR_Exon4_Rev	AATTAACGCAGGGTCAGTGG
Exon 5	PRLR_Exon5_For	AGCAAGGAAGCTCCATACCA
	PRLR_Exon5_Rev	GGAAGAAGGGTCAAGGGAAG
Exon 6	PRLR_Exon6_For	CACCTCATGTCACCCACTTG
	PRLR_Exon6_Rev	GTCCTGGGAGAGCTCTGATG
Exon 7	PRLR_Exon7_For	GCAGAGAGGGTGAAATGGTG
	PRLR_Exon7_Rev	TGGCCTAGGGAAAGATGCTA
Exon 8	PRLR_Exon8_For2	TATAGAGGGGCAGGGGACTT
	PRLR_Exon8_Rev2	GCCTCCATTTGATGGAAAGA
Exon 9	PRLR_Exon9_For2	GCAGCCATTTGGAAATAAGG
	PRLR_Exon9_Rev2	TAGCAGCAGCTAAGCGACAA
Exon 10	PRLR_3UTR_For1	TGACATCAGCCACTGTGAGG
	PRLR_3UTR_Rev2	CAGCCCAACTGGAGTCTGC
	PRLR_3UTR_For2	CCTATTTTCTGGCCAATGGA

PRLR_3UTR_Rev3	TGCAAAGGTTAAGCAACTGG
PRLR_3UTR_For4	GGCCTTCATGGTTTCGTATG
PRLR_3UTR_Rev5	TTTCACCCAGAGAAGTGAAAA
PRLR_3UTR_For5	TGAAATAAACCAGACATAGAAAGACAA
PRLR_3UTR_Rev6	CTGCTAGGGCAATGCTTCTC
PRLR_3UTR_For7	CACTGCTTGGAAATGCAGAA
PRLR_3UTR_Rev8	TTTGCACATCACTTTAGACTATGATTC
PRLR_3UTR_For8	TGGCTGAAGACTCAAACTGAA
PRLR_3UTR_Rev9	AGATTCCCCCTTTCAGTTGG
PRLR_3UTR_For9	ATGAGTCGGCCACAGGTTTA
PRLR_3UTR_Rev10	GCATGCAATTCAAAGCCATT
PRLR_3UTR_For11	CAACCGCTGAGTGGACTTTT
PRLR_3UTR_Rev12	TGAAGGTCTCAGAGCCAAAA
PRLR_3UTR_For12	CAGGAAAACCCTGCTGATGA
PRLR_3UTR_Rev14	CAGTTTTTGAACCACATAAAGCA
PRLR_3UTR_For12	CAGGAAAACCCTGCTGATGA
PRLR_3UTR_Rev15	TATGGAACCTTTGGCTGTCC
PRLR_3UTR_For15	TCTTTCGAAGCTGCTTATTGC
PRLR_3UTR_Rev16	GGCATTGTAACTAGTTCATGTAGAAA
PRLR_3UTR_For17	TTTCCCTAAAGCCCCTAGAAA
PRLR_3UTR_Rev18	AAAGCAGATGGCACCAGTGT
PRLR_3UTR_For18	TAACACATGCCTGGCTGAAA
PRLR_3UTR_Rev19	TCCCATATTGCAGGTGGATT
PRLR_3UTR_For20	ATCCCCTGGAGAAGGGATAG
PRLR_3UTR_Rev21	AGAATGCATGCCTGGAAAAA

Supplementary Table 7: *PRL* and *PRLR* oligonucleotide sequences

Primer sequences used to generate *PRL* and *PRLR* PCR amplicons for Sanger sequencing are indicated. Each ‘For’ and ‘Rev’ primer pair for a given amplicon are denoted by blue or pink background shading. Sequencing was conducted using the same primers used for amplification.
